# Nutrition and diet myths, knowledge and practice during pregnancy and lactation among a sample of Egyptian pregnant women: a cross-sectional study

**DOI:** 10.1186/s12884-024-06331-3

**Published:** 2024-02-16

**Authors:** Marwa Abdalla, Marwa M. Zein, Ahmed Sherif, Bassam Essam, Hend Mahmoud

**Affiliations:** 1 Kasr Al Ainy Hospital, Department of Obstetrics andGynecology, Cairo University, Cairo, 12256 Egypt; 2 Kasr Al Ainy Hospital, Department of Public Health, Cairo University, Cairo, 12256 Egypt; 3 General practitioner, Kasr Al Ainy Hospital, Cairo University, Cairo, Egypt

**Keywords:** Nutrition, Pregnancy, Lactating women

## Abstract

**Background:**

Globally, the burden of maternal malnutrition remains an enormous public health problem; malnourished pregnant women are at increased risk of having low-birth-weight (LBW) infants. Several reports suggest a possible association between malnutrition among lactating mothers and the production of smaller quantities of breast milk. Many women have incorrect nutrition knowledge during pregnancy due to false beliefs derived from popular practices. Our study was conducted to assess nutritional knowledge, myths, and practices among Egyptian women during pregnancy and lactation.

**Methodology:**

A cross-sectional study was conducted on 468 women attending antenatal clinics, including pregnant women and a small proportion of postpartum/lactating women who had delivered shortly before data collection. A pretested 2-page interview questionnaire was used to collect data from the study participants after written informed consent was obtained from them after clarification of the study’s aim. Obstetrics and gynecology experts collected the data from the females who agreed to participate in private and university hospital antenatal care clinics in Cairo, Egypt.

**Results:**

A total of 468 females completed the interview questionnaire. The mean knowledge score was 5 ± 3, with a median score of 5 and an IQR of 3–7, and the mean holding myths score was 3 ± 2, with a median score of 2 and an IQR of 2–4. Regarding the correct answers to the knowledge questions, more than 70% of the participants correctly answered that during the first six months of life, breast milk is the only food a baby requires, and less than 20% of themcorrectly answered that caffeine consumption could provoke premature birth. Regarding the holding myths questions, more than half of the participants heldthe myth that drinking moghat and helba increases the breast milk supply. We found that the most common source of knowledge during pregnancy and lactation among the participants was family and friends’ advice (60%), followed by others (doctors, previous education in school or university) (45%).

**Conclusion:**

Among a sample of Egyptian women, more than half held at least one myth about nutrition and diet during pregnancy and breastfeeding, so health education at antenatal outpatient clinics should be directed toward those myths to correct them. Older women with sufficient family income showed significantly higher knowledge scores than others.

**Supplementary Information:**

The online version contains supplementary material available at 10.1186/s12884-024-06331-3.

## Background

Globally, the burden of maternal malnutrition remains a substantial public health problem, with approximately 10% of women being undernourished [1]. A systematic review from Africa revealed that the overall pooled prevalence of maternal undernutrition was 20% [2].

The World Health Organization defines malnutrition as ‘the cellular imbalance between the supply of nutrients and energy and the body’sdemand for them to ensure specific functions, growth, and maintenance of the body [3].

Previous research has shown that pregnant women who are undernourished have a higher chance of giving birth to low birth weight babies. Numerous studieshave shown a connection between LBW and malnutrition, stunted growth and development, and higher rates of morbidity and mortality in children. This connection between LBW and poor health and nutritional outcomes later in life is also well documented [4]. Furthermore, there is evidence linking inadequate nutrition during pregnancy—particularly inadequacies in specific vitamins and minerals—to unfavorable outcomes for both the mother and the fetus. Preterm labor, inadequate anthropometric measurements, and birth asphyxia have all been associated with severe iron deficiency anemia. There are few studies on the effects of maternal malnutrition during lactation. According to a number of publications, there may be a connection between breastfeeding moms’ malnutrition and reduced milk supply as well as low levels of B vitamins, vitamin A, and important fatty acids [4].

Pregnant women’s malnutrition was caused by a number of factors that were collected from the research, including access to food, dietary taboos,poverty, and inadequate nutritional information—all of which continued to be significant obstacles for mothers and families [5].

Pregnant women’s nutritional knowledge and practice are important prerequisites for their proper nutritional intake. The antenatal period with opportunities for regular contact with health professionals appears to be the ideal time and setting to institute interventions that could maximize pregnant women’s outcomes and those of their babies by motivating them to make nutritional changes [6]. Many women are misinformed about pregnancy diet because of myths propagated by common practices. The conventional sources of knowledge regarding diet during pregnancy are insufficient to dispel the myths developed by traditions [7].

There is a clear need to provide dietary guidance during prenatal care and breastfeeding, and this guidance could be more effective if it takes myths into account. Because of the misconceptions and lack of information that surround diet in pregnancy and breastfeeding. This study was conducted to assess nutritional knowledge, myths, and practices among pregnant women. To our knowledge, no studies have been found in the literature measuring nutrition-related knowledge and myths among pregnant Egyptian women. The aim of our study was to explore the frequency of correct dietary knowledge and myths among pregnant women, to determine the frequency of correct dietary knowledge and myths among pregnant women, to define pregnant women´s source of dietary knowledge, and to find the relationship between correct dietary knowledge, myths, and sociodemographic characteristics of pregnant women.

## Methods

### Study design

An observational cross-sectional analytical study.

### Sample type

Convenient sample (easy access).

### Sample size and sampling technique

The sample size was 460, calculated using www.openepi.com, in view of a study performed in which 46% of the pregnant women had a myth score less than the mean [8]. The confidence interval was 95%, and the nonresponse rate was assumed to be 20% with a power of 80%. We used the following equation: Sample size n = [DEFF*Np(1-p)]/[(d2/Z21-α/2*(N-1) + p*(1-p)].

**The participants’ inclusion criteria were** Egyptian, pregnant, aged 21 years or older, married with no history of medical disorders, and willing to participate.

### Data collection tool

A pretested 2-page interview questionnaire was used to collect data from the study participants. It included four sections:

i) Questions assessing the social level of the family in Egypt: education, occupation, family size, residence, and income [9]. Age was collected as a self-reported variable and recorded in completed years as stated by participants at the time of interview. The observed clustering of ages at round numbers reflects a well-documented age-heaping phenomenon in self-reported survey data [10].
ii) Personal and obstetric history: age, age at first pregnancy, age at marriage, parity, gravidity, ANC provider facility, following regular ANC visits, and presence of chronic diseases.
iii) Knowledge and myths questions: Seven questions of myths and 10 questions of knowledge. These questions were obtained from a previous study conducted in Mexico [8]. With some modifications according to the Egyptian situation.

The questions were translated by two language experts into Arabic and back translated to English by another two independent language experts. Pilot testing of the final questionnaire was performed on 15 participants to ensure the clarity of the questions. The results of the pilot study were not added to the results. The Cronbach’s alpha reliability test was performed for the knowledge and myths question scales. The scales were internally consistent, with Cronbach’s alpha values of 0.819 and 0.745, respectively.

### Data collection technique

Obstetrics and gynecology experts collected the data from pregnant females who agreed to participate after written informed consent was obtained from them after clarification of the study’s aim in a private clinic and university hospital ANC clinic in Cairo, Egypt. The private clinic was open three days a week from 1 pm to 7 pm and served females of high socioeconomic level. The public ANC clinic of the university hospital was opened every day from 9 am to 3 pm except Friday and served females of middle and low socioeconomic levels. Five hundred pregnant women were assessed for eligibility, and 32 pregnant women were excluded: 14 did not meet the inclusion criteria, 6 did not consent and 12 refused to complete the questionnaire. Finally, 468 females completed the interview questionnaire. Although the primary inclusion criterion was pregnancy at the time of interview, a small proportion of participants (17 out of 468; 3.63%) were in the immediate postpartum period and were lactating at the time of data collection. These women were recruited from the same clinical settings due to high patient flow and the proximity of delivery to the interview. Their inclusion is consistent with the study title and objectives, which address nutrition during pregnancy and lactation.

### Statistical analysis

All the collected data were revised for completeness and logical consistency. The data were extracted from Google Forms to Microsoft Office Excel Software Program, 2019, and then transferred and analyzed into the Statistical Package of Social Science Software program, version 26 (SPSS) for statistical analysis. Mean, SD, median, and IQR were calculated for knowledge score and myths score, and then participants were categorized into 2 groups according to the median score for knowledge and for myths into above and below median score [8]. Comparisons between categories and sociodemographic charterers of the participants were performed using chi-square and Mann‒Whitney U tests accordingly, where a significant *p* value was *p* ≤ 0.05.

## Results

Of the 468 participants, 451 were pregnant and 17 were postpartum/lactating at the time of interview. The mean age at marriage was 21 ± 4 years, and the mean age at first pregnancy was 22 ± 4 years. As shown in Table 1, more than 80% of the females had less than a university education and more than 60% of their husbands had less than a university education. Regarding occupation, approximately 75% of the females were not working, and only 5% of their husbands were not working. Approximately 65% of these families’ income was just enough, and less than a quarter of these families (23.1%) were living in houses with a crowding index greater than 2.

**Table 1:** Sociodemographic characteristics of Egyptian pregnant female participants (*n* = 468):

		Count	Column N %
Wife education	below university	380	81.2%
	above university	88	18.8%
Husband education	below university	303	64.7%
	above university	165	35.3%
Occupation (Wife)	Not working	348	74.4%
	working	120	25.6%
Occupation (Husband)	Not working	25	5.3%
	working	443	94.7%
Average family income	It is not enough, and we have a big debt	55	11.8%
	It is not enough, and we have a small debt	88	18.8%
	just enough	303	64.7%
	enough	22	4.7%
Residence	Urban	293	62.6%
	Rural	175	37.4%
Crowding index (CI)*	<=2 (not crowded)	360	76.9%
	> 2 (crowded)	108	23.1%
Regular ANC	No	71	15.2%
	Yes	397	84.8%
Where do you do ANC	Public/governmental hospital	70	17.6%
	Private hospital	277	69.8%
	Primary health care	49	12.3%
	Others	1	0.3%

*CI = number of individuals in a house/number of bedrooms [11]

The mean knowledge score was 5 ± 3 with a median score of 5 and an IQR of 3–7, and the mean holding myths score was 3 ± 2 with a median of 2 and an IQR of 2–4.

**Fig. 1:**
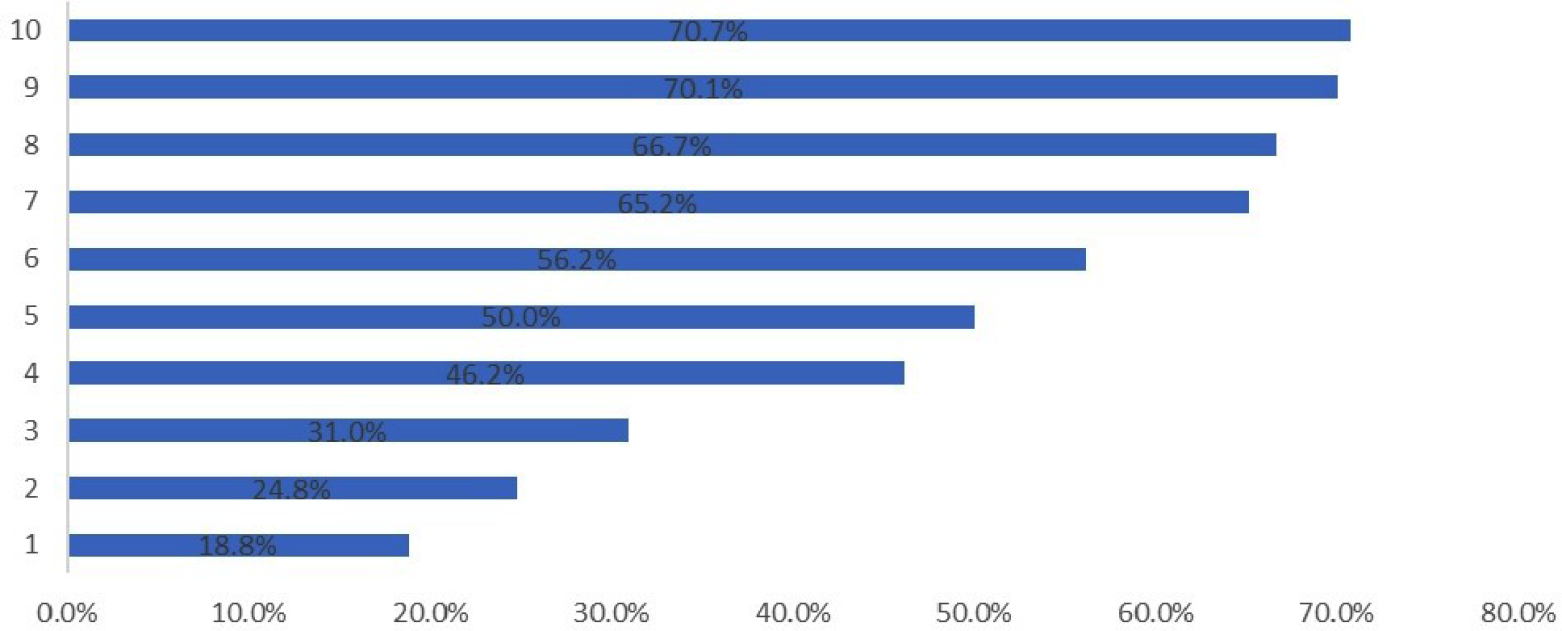
Percentage distribution of females answering the knowledge statements correctly 1-Caffeine consumption provokes premature birth 2-Folic acid intake should begin before not only during pregnancy 3-Gestational diabetes increases future risk of type 2 diabetes 4-The mother requires an adequate increased energy intake during pregnancy and nursing 5-Obesity during pregnancy can cause hypertension and the risk of preeclampsia 6-A mother should consume approximately 3 L of water per day while nursing 7-A healthy diet and lifestyle during pregnancy prevent future diseases in a child 8-For successful breastfeeding, a baby must latch on to the nipple and use suction to feed 9-The fetus receives vitamins, proteins, and minerals from what the mother eats 10-During the first six months of life, breast milk is the only food a baby requires

Figure 1 illustrates the correct answers to the knowledge questions. More than 70% of the participants correctly answered that breast milk is the only food a baby requires during the first six months of life, and less than 20% answered correctly that caffeine consumption provokes premature birth.

**Fig. 2:**
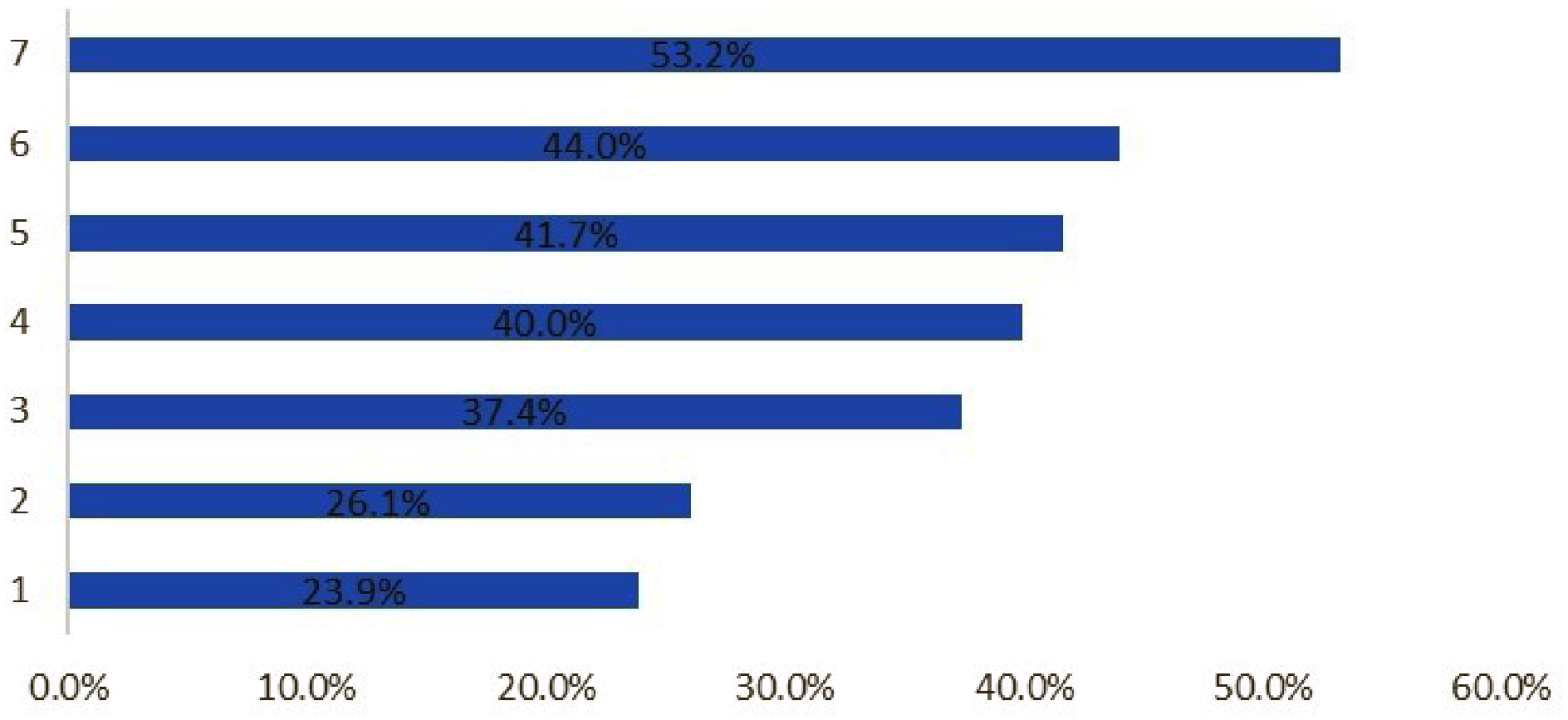
Percent distribution of participants who held myths 1-Vomiting cannot be controlled during pregnancy 2-It is impossible to recover one’s prebaby weight after childbirth 3-You cannot exercise during pregnancy 4-An angry mother should not nurse her baby 5-You must eat for two during pregnancy 6-Not satisfying cravings leave a mark on the body of the newborn 7-You should drink moghat and helba to increase the breast milk supply

Figure 2 illustrates the holding myths questions; more than half of the participants held the myth that drinking moghat and helba increases the breast milk supply.

**Fig. 3:**
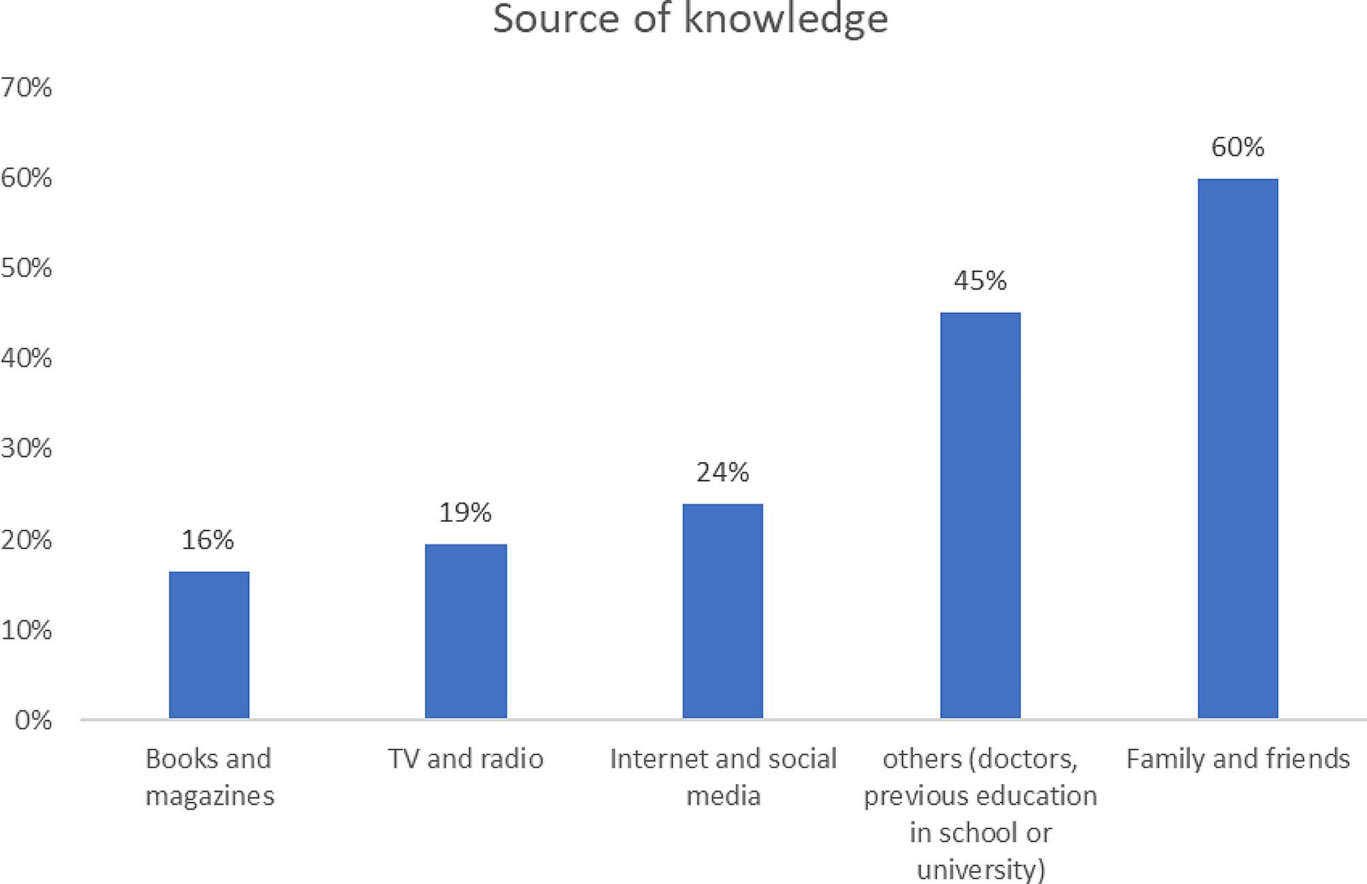
Percent distribution of the main source of knowledge the participants trusted for knowledge related to pregnancy and lactation

Figure 3 shows that the most common sources of knowledge regarding pregnancy and lactation among the participants were family and friends’ advice (60%), followed by others (doctors, previous education in school or university) (45%).

When we compared the knowledge score and sociodemographic characteristics of the participants, we found that participants with older age and sufficient family income showed significantly higher knowledge scores than others (*p* value 0.002, < 0.001, respectively). No other significant difference was found between the sociodemographic characteristics of the participants, and either the knowledge or the myths scores (Table 2). It is also shown in Table 2 that women with higher median knowledge were more common among women with higher gravida when the wife and husband were educated more than a university education, and among those who lived in urban residences. The higher median holding myths were among women with low gravida, educated below university for wife and husband, who live in rural residences, and with a higher crowding index.

**Table 2:** Comparison between sociodemographic characteristics of the participants and the knowledge, and myths scores

Sociodemographic characters		Knowledge score					Holding myths score				
		Below median score (*n* = 256)		above median score (*n* = 212)		*p* value	below median score (*n* = 337)		above median score (*n* = 131)		*p* value
Age (years) mean ± sd, median (IQR)		28 ± 6	28 (23–32)	30 ± 7	30 (25–35)	0.002*	29 ± 6	29 (24–33)	29 ± 7	29 (25–34)	0.894
Age at first pregnancy (years) mean ± sd, median (IQR)		22 ± 4	21 (18–25)	22 ± 5	21 (19–25)	0.397	22 ± 5	21 (18–25)	22 ± 4	21 (19–25)	0.732
Age at marriage (years) mean ± sd, median (IQR)		21 ± 4	20 (17–24)	21 ± 4	20 (18–24)	0.284	21 ± 4	20 (17–24)	21 ± 4	20 (18–24)	0.929
Gravida mean ± sd, median (IQR)		3 ± 2	3 (2–4)	4 ± 2	3 (2–5)	0.175	4 ± 3	3 (2–4)	3 ± 2	3 (2–4)	0.974
Parity mean ± sd, median (IQR)		2 ± 2	2 (1–3)	2 ± 2	2 (1–3)	0.356	2 ± 2	2 (1–3)	2 ± 2	2 (1–3)	0.795
wife education n (%)	below university	216	84.4%	164	77.4%	0.053	270	80.1%	110	84.0%	0.338
	above university	40	15.6%	48	22.6%		67	19.9%	21	16.0%	
husband education n (%)	below university	171	66.8%	132	62.3%	0.307	216	64.1%	87	66.4%	0.638
	above university	85	33.2%	80	37.7%		121	35.9%	44	33.6%	
Wife Occupation n (%)	Not working	189	73.8%	159	75.0%	0.773	252	74.8%	96	73.3%	0.648
	working	67	26.2%	53	25.0%		85	25.2%	35	26.7%	
Husband Occupation n(%)	Not working	16	6.3%	9	4.2%	0.337	19	5.6%	6	4.6%	0.674
	working	240	93.8%	203	95.8%		318	94.4%	125	95.4%	
Average family income n (%)	It is not enough, and we have a bigdebt	44	17.2%	11	5.2%	< 0.001*	39	11.6%	16	12.2%	0.393
	It is not enough, and we have a small debt	60	23.4%	28	13.2%		68	20.2%	20	15.3%	
	just enough	149	58.2%	154	72.6%		214	63.5%	89	67.9%	
	enough	3	1.2%	19	9.0%		16	4.7%	6	4.6%	
Residence n (%)	Urban	156	60.9%	137	64.6%	0.412	215	63.8%	78	59.5%	0.851
	Rural	100	39.1%	75	35.4%		122	36.2%	53	40.5%	
Crowding index n (%)	<=2	197	77.0%	163	76.9%	0.986	260	77.2%	100	76.3%	0.591
	> 2	59	23.0%	49	23.1%		77	22.8%	31	23.7%	

*Significant

Table 3 shows the practice of nine knowledge and myths statements. Approximately 45% of the women practiced drinking moghat and helba to increase their breast milk supply, and 40% satisfied the cravings during pregnancy and consumed more than 3 L of water per day while nursing. More than half were practicing breastfeeding for their infants and ensuring good latch on of the baby.

**Table 3:** Practices toward knowledge and myths among participants (*n* = 468)

	I already do that	I would do that	I do not do that	I have not done that	I would not do that
1- You must eat for two during pregnancy	150 (32.1)	28 (6.0)	143 (30.6)	143 (30.6)	4 (0.9)
2. You should drink moghat and helba to increase the breast milk supply	210 (44.9)	69 (14.7)	109 (23.3)	69 (14.7)	11 (2.4)
3- you should satisfy cravings for certain food during pregnancy	187 (40.0)	49 (10.5)	147 (31.4)	81 (17.3)	4 (0.9)
4- You should exercise during pregnancy	108 (23.1)	71 (15.2)	170 (36.3)	116 (24.8)	3 (0.6)
5-An angry mother shouldn’t nurse her baby	121 (25.9)	49 (10.5)	175 (37.4)	97 (20.7)	26 (5.6)
6- Folic acid intake should begin before not only during pregnancy	47 (10.0)	46 (9.8)	152 (32.5)	218 (46.6)	5 (1.1)
7- A mother should consume approximately 3 L of water per day while nursing	186 (39.7)	90 (19.2)	119 (25.4)	63 (13.5)	10 (2.1)
8-I should breastfeed my baby	265 (56.6)	101 (21.6)	59 (12.6)	37 (7.9)	6 (1.3)
9- For successful breastfeeding, a baby must latch on to the nipple and use suction to feed	258 (55.1)	85 (18.2)	78 (16.7)	25 (5.3)	22 (4.7)

## Discussion

Pregnant women must maintain a sufficient nutritional level for both their health and the success of their pregnancy. Due to increased nutritional requirements, pregnancy is a critical period for meeting the body’s demand for macro/micronutrients. Worldwide, 53.8 million pregnant women suffer from common micronutrient deficiencies such as anemia and vitamin A deficiency (VAD). In addition to inadequate nutrition, social and psychological factors, women’ nutritional awareness, and biological changes that affect how they perceive their eating habits during pregnancy all have an impact on maternal malnutrition [3].

Breastfeeding is very important to human infants (aged ≤ 12 months) and young children (aged 12–36 months), as it promotes healthy brain development and is essential for preventing the triple burden of malnutrition, infectious diseases, and mortality, while also reducing the risk of obesity and chronic diseases in later life in low-income and high-income countries alike. Breastfeeding also helps to protect the mother against chronic diseases, including breast and ovarian cancers, type 2 diabetes, and cardiovascular disease [12].

Antenatal care refers to the follow-up care that pregnant women receive to ensure the greatest possible health for both the mother and the fetus. This care includes education, counseling, screening, and treatment. One of the most important things in preventing women’s deaths from avoidable causes of maternal death is health awareness [13].

Education is an important component of prenatal care, particularly for women who are pregnant for the first time. Numerous studies have revealed that educated women experience better pregnancy outcomes than uneducated women and that prenatal education can help to lower the risk of difficulties during pregnancy and delivery [14].

Comprehensive and integrated antenatal care should include nutrition education, to obtain optimal health status for both the mother and child [15].

### Myths about nutrition and diet during pregnancy and lactation

The present study is one of the first to provide information on the myths and knowledge of pregnant Egyptians regarding nutrition and diet during pregnancy and lactation. We found that more than half of the participants held at least one myth about nutrition and diet during pregnancy and breastfeeding. 44% believed that not satisfying cravings leave a mark on the body of the newborn during pregnancy, and 40% of our participants would act upon this myth regardless of their educational level, while another study on pregnant Mexicans found that, lower educational attainment was associated with.

holding and acting upon myth [8]. This study also found that younger participants, The belief that “a frightened or angry mother should not nurse a baby” was most prevalent among women with the lowest levels of education, the lowest socioeconomic status, and fewer prior pregnancies, whereas in our study, 40% of the participants believed that, but only 25.9% believed that [8]. Weir.

Z. et al. performed a qualitative study of the myths of overweight and obese pregnant women and reported that the myth “you cannot exercise during pregnancy” was associated with a low socioeconomic level while our study revealed that, 37.4% believed that and 23.1% indeed were not practicing exercise regardless of their socioeconomic level [16]. According to Marshall et al., pregnant women in a rural community in the southeast United States typically do not engage in physical activity because they feel that their everyday activities provide enough exercise. While some felt that physical exercise puts both mother and child at risk for injury, others felt that rest is more vital than physical activity [17]. In contrast to pregnant Maasai women in Tanzania, during the second and third trimesters, they will gradually increase the amount of daily chores they have to perform until they become exhausted. They will also neglect their prenatal checkups in order to prepare for their postnatal period, during which they will remain in their homes for three months [18]. Nonetheless, Atif et al. found that 76.4% of respondents in Pakistan thought that light to moderate weight lifting was a cause of miscarriage [19].

During lactation, 53.2% thought that drinking moghat and helba could increase the breast milk supply and 44.9% were already practicing that.

### Knowledge

In our study, participants with older age and with enough family income showed significantly higher knowledge scores than others, and the reason may be due.

to the fact that older women have more experience. Generalized Egyptian beliefs were prevalent among our study population, which included the idea that the fetus receives vitamins, proteins, and minerals from the mother’s diet and that breast milk is the only nourishment a baby needs during the first six months of life. However, in a community-based qualitative cross-sectional study, that was conducted between April and May 2022 among the rural Acholi communities in Northern Uganda or newborns, they found that most mothers believed that breast milk is given exclusively in the first two months. However, some mothers, introduce sugar solution, black tea, and soup immediately after birth [20].

We also want to draw attention to the fact that, although two-thirds of study women from the Middle East used folic acid, only 47% of our participants took it before or throughout pregnancy [21].

In January/February 2022, a cross-sectional questionnaire-based real-world research was carried out in Vietnam with 200 pregnant women and 100 preconception women. Out of 300 respondents, 62% had scores indicating a high likelihood of limited health literacy, 33% had scores indicating possibly limited health literacy, and only 5% (16 respondents) had adequate health literacy. Only 23% of respondents with limited health literacy were currently using folic acid/folate supplements, whereas 39% of those with possibly limited health literacy were using folic acid/folate supplements (*p* < 0.05). This indicates that folic acid administration before and during pregnancy is insufficient even in other countries [22].

Other studies reported that most women know that they should take folic acid but do not know exactly how folic acid could benefit them and their babies (e.g., prevention of neural tube defects) [23].

We did notice and would like to highlight, that only 31% of our participants knew that gestational diabetes increases the future risk of type 2 diabetes. Another study showed that 80% of their study participants knew about this increased risk [8], which reflects the magnitude of the lack of knowledge among Egyptian women. We think that this lack of knowledge may prevent women from seeking professional advice for improving their diet and lifestyle on time to delay or prevent the onset of type 2 diabetes mellitus.

### Sources of nutritional knowledge

Our research revealed that the participants’ most frequent sources of information about pregnancy and lactation were advice from family and friends (60%) and others (doctors, previous education in school or university) (45%) respectively.

However, according to a different Vietnamese survey, healthcare professionals (77%), family or friends (47%), and websites (36%), were the most reliable sources of information about nutrition [22].

### Implications

Generally, pregnant, and lactating women tend to modify their diet according to myths and knowledge transmitted from their family and friends, who were the main source of knowledge among our participants to improve their health and that of their children. However, they often implement these modifications without the guidance of a nutrition or health specialist, as reported by Pinheiro [24]. This circumstance offers perinatal healthcare providers an opportunity to enhance the nutrition and gestational weight gain of their patients, particularly younger patients with less expertise, by implementing appropriate intervention programs. It is crucial that dieticians and other medical experts take on the responsibility of dispelling misconceptions about nutrition and diet during pregnancy and lactation. Finally, it is essential to highlight that nutritional guidance for pregnant women will require not only the transmission of knowledge but also the interpretation of information within the context of certain culturally rooted beliefs [25].

Our study’s limitations were its convenient sample and that it is a single-center study, so a randomized study that includes different areas in Egypt with large is needed for a better assessment of nutritional knowledge, myths, and practices among Egyptian women during pregnancy and lactation.

## Conclusion

The findings reflect nutritional knowledge, myths, and practices among Egyptian women during pregnancy and lactation. More than half held at least one myth about nutrition and diet during pregnancy and breastfeeding. Therefore, health education at antenatal outpatient clinics should be directed toward correcting these myths to improve the nutritional status of pregnant women in Egypt. Older women with sufficient family income showed significantly higher knowledge scores than others.

## Electronic supplementary material

Below is the link to the electronic supplementary material.

Supplementary Material 1

Supplementary Material 2

## Data Availability

The datasets used and analyzed during the current study are attached in the supplementary material section.

## Abbreviations

ANC: Antenatal care
LBW: Low-birth-weight
