# Correction: Nutrition and diet myths, knowledge and practice during pregnancy and lactation among a sample of Egyptian pregnant women: a cross-sectional study

**DOI:** 10.1186/s12884-026-09140-y

**Published:** 2026-07-10

**Authors:** Marwa Abdalla, Marwa M. Zein, Ahmed Sherif, Bassam Essam, Hend Mahmoud

**Affiliations:** 1 Department of Obstetrics and Gynecology, Kasr Al Ainy Hospital, Cairo University, Cairo, 12256 Egypt; 2 Department of Public Health, Kasr Al Ainy Hospital, Cairo University, Cairo, 12256 Egypt; 3 General practitioner, Kasr Al Ainy Hospital, Cairo University, Cairo, Egypt

**Correction: BMC Pregnancy Childbirth 24**,** 140 (2024)**

**https://doi.org/10.1186/s12884-024-06331-3**

Following publication of the original article [1], the authors reported an error in Abstract, maintext and reference.

**Abstract**

In the Methods section of the Abstract, the sentence reading:

A pretested 2-page interview questionnaire was used to collect data from the study participants after written informed consent was obtained from them after clarification of the study’s aim. Obstetrics and gynecology experts collected the data from pregnant females who agreed to participate in private and university hospital antenatal care clinics in Cairo, Egypt.

Should be updated to:

A cross-sectional study was conducted on 468 women attending antenatal clinics, including pregnant women and a small proportion of postpartum/lactating women who had delivered shortly before data collection. A pretested 2-page interview questionnaire was used to collect data from the study participants after written informed consent was obtained from them after clarification of the study’s aim. Obstetrics and gynecology experts collected the data from the females who agreed to participate in private and university hospital antenatal care clinics in Cairo, Egypt.

In the results section of the abstract, the sentence reading:

A total of 468 pregnant females completed the interview questionnaire.

Should be updated to:

A total of 468 females completed the interview questionnaire.

**Methods**

In the Data collection tool section, after the sentence reading:

“Questions assessing the social level of the family in Egypt: education, occupation, family size, residence, and income [9].”

The following sentence will be added:

“Age was collected as a self-reported variable and recorded in completed years as stated by participants at the time of interview. The observed clustering of ages at round numbers reflects a well-documented age-heaping phenomenon in self-reported survey data [10].”

In the data collection technique section, the sentence reading in the last sentence:

Finally, 468 pregnant women were included in the study and completed the questionnaire.

Should be updated to:

Finally, 468 females completed the interview questionnaire. Although the primary inclusion criterion was pregnancy at the time of interview, a small proportion of participants (17 out of 468; 3.63%) were in the immediate postpartum period and were lactating at the time of data collection. These women were recruited from the same clinical settings due to high patient flow and the proximity of delivery to the interview. Their inclusion is consistent with the study title and objectives, which address nutrition during pregnancy and lactation.

**Results**

The sentence reading in the first sentence of the results:

A total of 468 pregnant women completed the interview questionnaire.

should be updated to:

Of the 468 participants, 451 were pregnant and 17 were postpartum/lactating at the time of interview.

**Conclusion**

The Conclusions paragraph reading:

Among a sample of Egyptian women, more than half held at least one myth about nutrition and diet during pregnancy and breastfeeding, so health education at antenatal outpatient clinics should be directed toward those myths to correct them, and improve the nutritional status of pregnant Egyptians. Older women with sufficient family income showed significantly higher knowledge scores than others.

Should be replaced with:

The findings reflect nutritional knowledge, myths, and practices among Egyptian women during pregnancy and lactation. More than half held at least one myth about nutrition and diet during pregnancy and breastfeeding. Therefore, health education at antenatal outpatient clinics should be directed toward correcting these myths to improve the nutritional status of pregnant women in Egypt. Older women with sufficient family income showed significantly higher knowledge scores than others.

**Reference**

The following reference should be added:

Heeringa SG, West BT, Berglund PA. Applied survey data analysis. Chapman and Hall/CRC; 2017.

This should be added as reference number [10], and all subsequent references should be renumbered accordingly

The original article has been corrected.
