# Supplementary figures and images for: Nutrition and diet myths, knowledge and practice during pregnancy and lactation among a sample of Egyptian pregnant women: a cross-sectional study
Source: BMC Pregnancy Childbirth. 2024 Feb 16;24:140. doi: 10.1186/s12884-024-06331-3 (PMC10870649; doi:10.1186/s12884-024-06331-3)

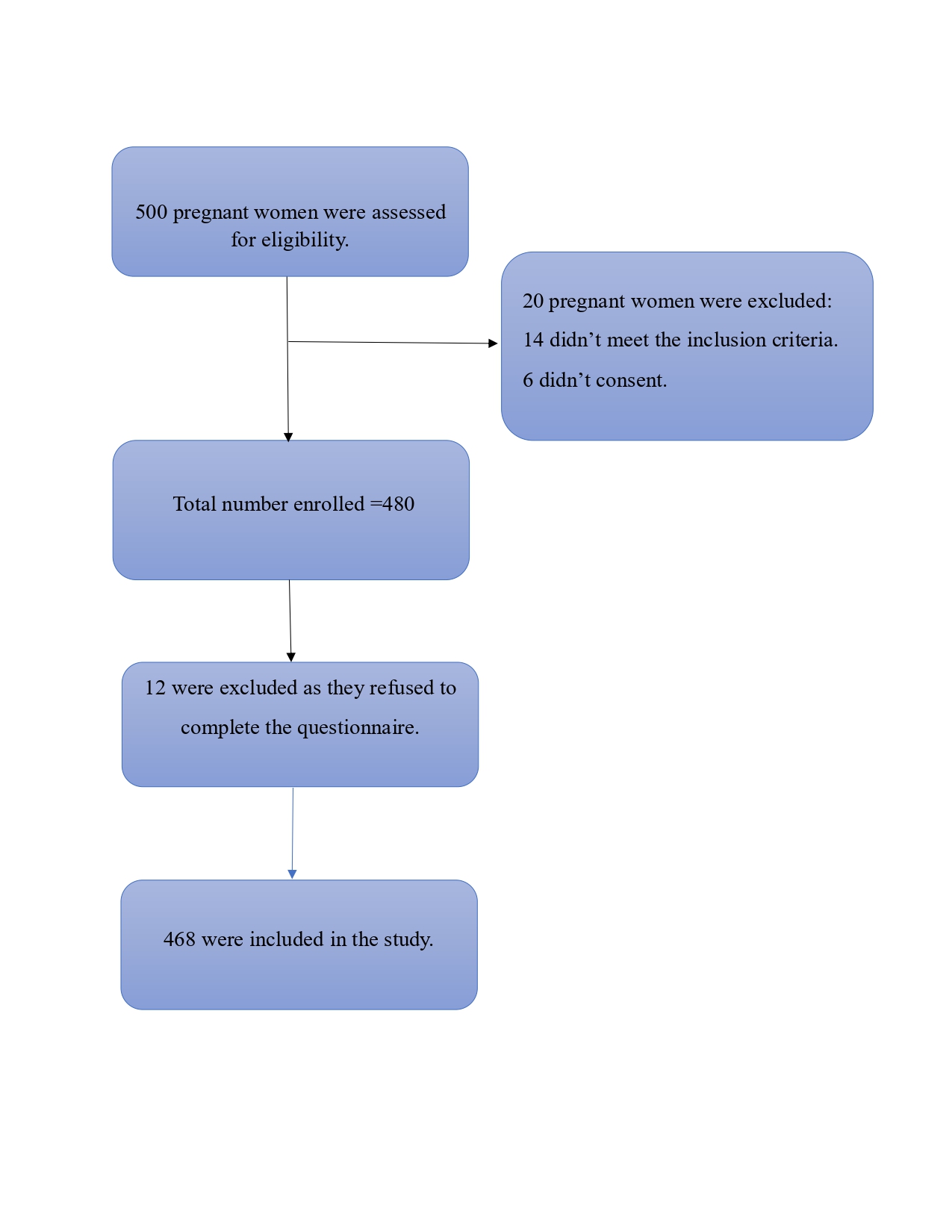


Consort flow-chart of the recruitment process

Supplement: Supplementary file 1 — Supplementary Material 1 [file 12884_2024_6331_MOESM1_ESM.docx]
